# Successful treatments of idiopathic retinal vasculitis, aneurysms, and neuroretinitis (IRVAN) syndrome–related macular edema and exudation by intravitreal aflibercept injection in a senile patient: A case report

**DOI:** 10.1097/MD.0000000000039768

**Published:** 2024-09-27

**Authors:** Hsiao-Fan Tung, Jian-Sheng Wu, San-Ni Chen

**Affiliations:** a Department of Ophthalmology, China Medical University Hospital, China Medical University, Taichung, Taiwan; b Department of Ophthalmology, Changhua Christian Hospital, Changhua, Taiwan; c Department of Optometry, Da-Yeh University, Changhua, Taiwan; d School of Medicine, Chung-Shan Medical University, Taichung, Taiwan.

**Keywords:** idiopathic retinitis, vasculitis, aneurysms, neuroretinitis syndrome, intravitreal aflibercept injection, intravitreal anti-VEGF injection, IRVAN syndrome

## Abstract

**Rationale:**

The prevalence of idiopathic retinitis, vasculitis, aneurysms, and neuroretinitis (IRVAN) syndrome is <1/1,000,000, and it tends to occur in young women. Panretinal photocoagulation, focal laser photocoagulation, peripheral cryopexy, vitrectomy, and corticosteroid therapy are the traditional treatments. We reported a case of a senile patient with IRVAN syndrome who presented with severe exudation in both eyes and macular edema in the left eye, successfully treated by serial intravitreal aflibercept injections.

**Patient Concerns:**

A 77-year-old Taiwanese woman complained of progressive blurred vision in the left eye and ocular examinations revealed a visual acuity of 20/125 in the left eye.

**Diagnosis:**

Indirect fundoscopy and fluorescein angiography showed bilateral multiple aneurysms, vasculitis, optic nerve staining, and neovascularizations. In addition, optical coherence tomography demonstrated macular edema with subretinal fluid and exudations in the left eye.

**Interventions:**

Monthly intravitreal injections of antivascular endothelial growth factor with aflibercept 2.0 mg were administered in the left eye.

**Outcomes:**

The visual acuity in the left eye improved to 20/50 after 18 months of treatment. Macular edema and subretinal fluid regressed. Furthermore, vessel leakage and optic disc staining also improved.

**Lessons:**

This case is the first to demonstrate successful treatment of IRVAN syndrome-related central macular edema and exudations, in the absence of neovascularization, using a series of intravitreal aflibercept injections as monotherapy.

## 1. Introduction

Idiopathic retinitis, vasculitis, aneurysms, and neuroretinitis (IRVAN) syndrome is first mentioned in 1995 by Chang et al.^[1]^ The prevalence is <1/1,000,000, and the disease tends to happen in young women. As far as current understanding, the diagnosis of the disease is mainly according to a group of clinical features, including retinal vasculitis, aneurysmal dilations at arterial bifurcations, neuroretinitis, peripheral capillary nonperfusion, retinal neovascularization, and macular exudation; the first 3 are the major and the last 3 are the minor criteria.^[1,2]^ Besides, optical coherence tomography (OCT) and fluorescein angiography (FA) also play an important role in disease diagnosis. Panretinal photocoagulation (PRP), focal laser photocoagulation, peripheral cryopexy, vitrectomy, and corticosteroid therapy are the common choices for treatments traditionally. Nowadays, intravitreal injection (IVI) of anti-vascular endothelial growth factor (anti-VEGF) with bevacizumab or ranibizumab was announced and reported with good efficacy in several cases.^[3–6]^

We reported a senile patient of IRVAN syndrome who presented with severe exudation in both eyes and macular edema in the left eye, which was successfully treated by a serial intravitreal aflibercept injection.

## 2. Case presentation

**Figure 1.**
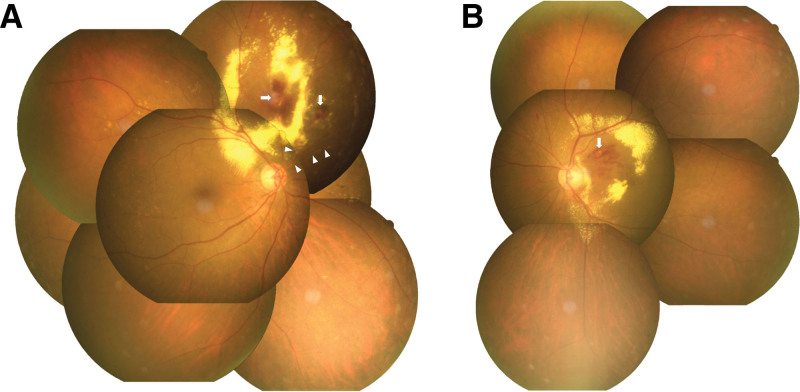
Fundus photography of the bilateral eyes when the patient first visited. Aneurysms with exudates were found in bilateral eyes. There was obvious vessel sheathing in the superior nasal retina in the right eye. (A) right eye, (B) left eye (White arrow: macroaneurysm and white arrowhead: vessel sheathing.)

**Figure 2.**
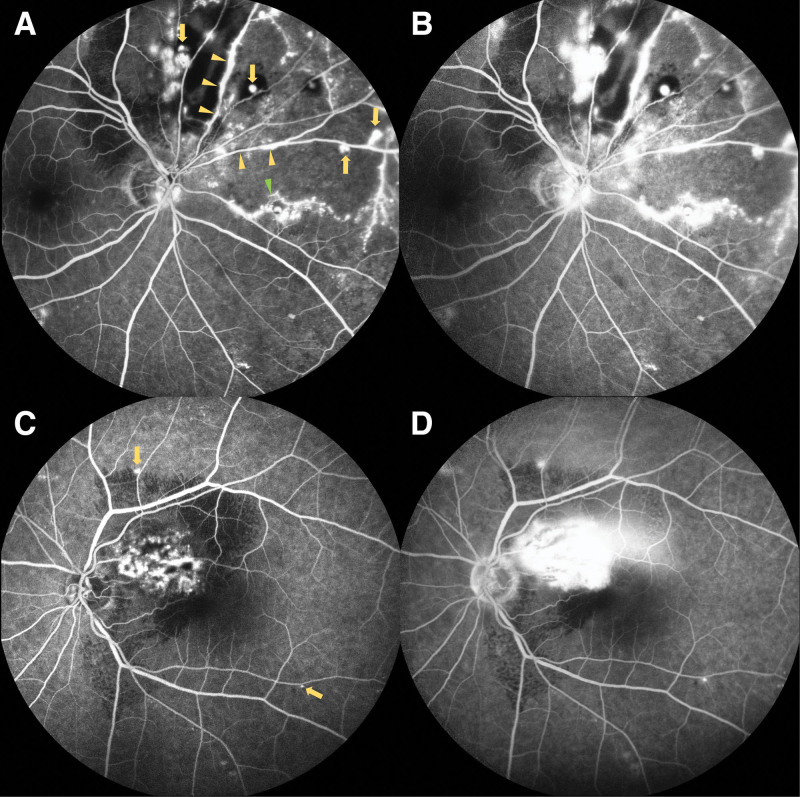
Fluorescein angiography when the patient was first visited. (A) Early stage in the right eye. (B) Late stage in the right eye. (C) Early stage in the left eye. (D) Late stage in the left eye. (A) and (B) Multiple aneurysms, obliteration, and leakage of retinal vessels with nonperfusion areas mainly over superior and nasal sides. Besides, neovascularization and optic nerve head staining were also found. In (C) and (D), obvious vessels’ leakage was noted over the superior macular area. (Yellow arrow: aneurysms; yellow arrowhead: hyperfluorescence of the vessel walls; and green arrowhead: neovascularization.)

**Figure 3.**
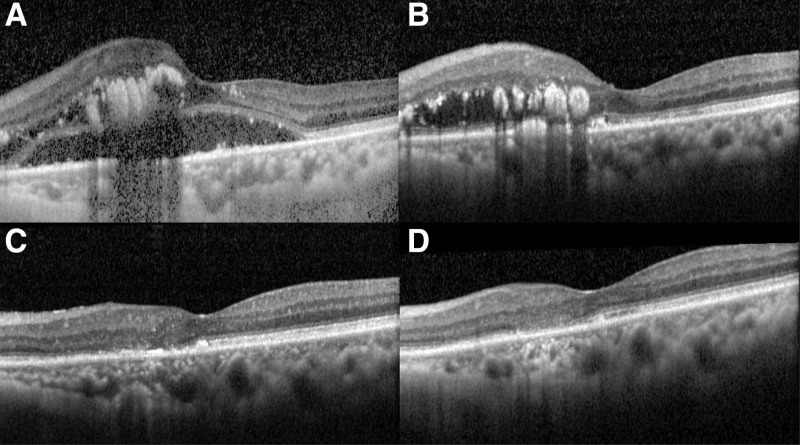
(A) The optical coherence tomography when the patient first visited. Severe macular edema and exudations with subretinal fluid. (B) 6, (C) 12, (D) 18 months after intravitreal injection with aflibercept every 4 weeks, which revealed significant regression of macular edema.

A 77-year-old Taiwanese woman consulted our ophthalmology clinic with the chief complaint of progressive blurred vision in the left eye for 2 months. She has underlying diseases of hypertension, type 2 diabetes mellitus, and hyperlipidemia, all were under medical treatment. On ocular examinations, the best-corrected visual acuity (BCVA) was 20/40 in the right eye and 20/125 in the left eye. In the right eye, indirect fundoscopy revealed multiple aneurysms with exudation over the suprapapillary area and retinal vascular sheathing over the nasal upper side (Fig. 1A). Not only multiple aneurysms but also obliteration and leakage of retinal vessels with nonperfusion areas, neovascularization, and optic nerve head staining were disclosed by FA (Fig. 2A and 2B). On the other hand, in the left eye, an aneurysm over the superior nasal macula with peripheral exudates and some peripapillary exudation were found in the fundoscopic exam (Fig. 1B). Vascular leakage with severe macular edema accompanied by subretinal fluid and exudation was showed in FA and OCT (Figs. 2C and 2D and 3A). The above findings match the diagnostic criteria of IRVAN syndrome.

**Figure 4.**
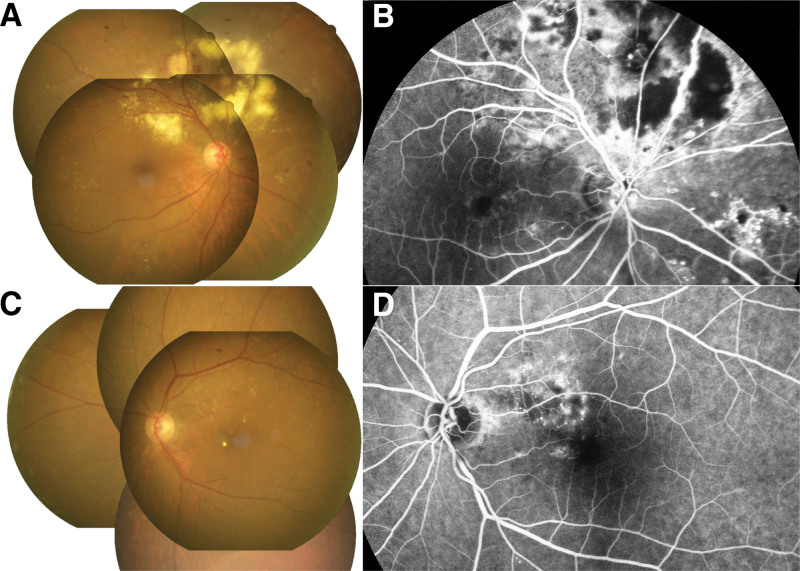
Eighteen months after treatments. (A) and (B) Though nonperfusion areas enlarged and vessel leakages got worse after 1 year after observation, the severity became stable after FRP twice on the aneurysms. (C) and (D) Vascular leakage improved and the amount of exudation diminished obviously in the left eye. FRP = focal retinal photocoagulation.

In the left eye, we arranged monthly IVI of anti-VEGF with aflibercept 2.0 mg immediately after the disease was diagnosed. After 6 times of intravitreal aflibercept injections, the subretinal fluid was remitted and the BCVA got 20/100 (Fig. 3B). Under the regular treatments, macular edema and the exudates decreased gradually. The exudates diminished obviously after 1 year of injections, and the BCVA achieved 20/65 (Fig. 3C). We kept the therapy for 18 months when the macular edema and exudates complete remission (Fig. 3D). At the time, BCVA improved and got 20/50. OCT exam revealed the inner segment/outer segment junction disruption over the perifoveal area, which might result in some limitations of visual improvement. Besides, in the fundoscopy and FA, vessel leakage and optic disc staining also improved a lot (Fig. 4). The patient was regularly followed up at our clinic, and no more macular edema or subretinal fluid was found after we held the injection.

In the right eye, due to no posterior pole involvement, the visual acuity was not affected. No IVI was suggested. After following for a year, the nonperfusion areas enlarged, and vessel leakages got more severe. Thus, we chose focal laser photocoagulation for the aneurysms. The laser was done twice, and the condition became stable after the treatments.

## 3. Discussion

IRVAN syndrome was reported to be present mostly in young female patients. However, more and more patients with senior ages were reported in recent series.^[3,7]^ According to the staging system proposed by Samuel et al,^[2]^ IRVAN syndrome is staged into 5 stages: macroaneurysms, exudation, neuroretinitis, and retinal vasculitis; capillary nonperfusion (angiographic evidence); posterior segment neovascularization of disc or elsewhere and/or vitreous hemorrhage; anterior segment neovascularization; and neovascular glaucoma. In this report, we presented a case of stage 2 IRVAN in a senile woman with multiple systemic diseases, including diabetes mellitus, hypertension, and hyperlipidemia. From the FA, there were no signs of diabetic retinopathy outside the area of IRVAN, which precludes the diagnosis of diabetic retinopathy. Besides, the multiple arterial involvement also makes the diagnosis of branch retinal vein occlusion unlikely. In contrast, the fusiform and saccular aneurysm along with nonperfusion area and heavy exudation more coped with the diagnosis of IRVAN syndrome. Though macular exudation is a typical finding in IRVAN, the heavy exudation in our case may be partially attributed to the underlying disease of diabetes mellitus, hypertension, and hyperlipidemia, which would aggravate the originally compromised vasculature brought by IRVAN. Up until now, there were only a few cases of IRVAN syndrome–related central macular edema and exudations managed by antivascular growth factors reported.^[6,7]^ In this case, we treated the left eye with aflibercept with great efficacy. Subretinal fluid diminished after 6 months, macular edema regressed after 1 year, and all exudates resolved after one and a half years of regular injections. Besides, the condition of the posterior pole in the left eye remained stable after we stopped the injection for >6 months. Except for the good efficacy of aflibercept, early diagnosis and treatments also played an important role in the successful outcome.

Due to the important effects of suppressing the inflammatory and angiogenesis process, anti-VEGF agents are widely used to treat various kinds of retinal diseases nowadays.^[8]^ Though the actual etiology of IRVAN syndrome has not been proved, the presentations of the disease are mainly by inflammation of the retinal vessels and the nonperfusion of the retina, which might be the indication of intravitreal anti-VEGF agent injection. In 2010, Akesbi et al^[3]^ reported a favorable result of neovascular regression after IVI bevacizumab followed by PRP and vitrectomy in a stage 4 patient. Karagiannis et al^[4]^ also presented a grateful outcome in a stage 3 case who underwent IVI ranibizumab, PRP, and vitrectomy. In 2018, Massicotte et al^[7]^ first described the successful treatment of macular exudation in IRVAN syndrome without neovascularization by IVI bevacizumab. Currently, the only report about applying aflibercept in IRVAN syndrome was announced by Jones et al.^[5]^ In this case, marked improvement in cystoid macular edema was found after shifting from the IVI bevacizumab to IVI aflibercept.^[5]^

The above cases and our patient gave us good examples and experiences in treating IRVAN syndrome with intravitreal anti-VEGF agent injection. Though larger trials in the future are needed to ensure the efficacy of different kinds of anti-VEGF agents in this special disease, the effectiveness of aflibercept in IRVAN is worth expecting.

In conclusion, this case first demonstrated a successful treatment by a serial of intravitreal aflibercept injection as monotherapy in IRVAN syndrome–related central macular edema and exudations in the absence of neovascularization.

## Author contributions

**Data curation:** Hsiao-Fan Tung, San-Ni Chen.

**Formal analysis:** Hsiao-Fan Tung.

**Methodology:** Hsiao-Fan Tung, Jian-Sheng Wu, San-Ni Chen.

**Visualization:** Hsiao-Fan Tung.

**Writing – original draft:** Hsiao-Fan Tung.

**Investigation:** Jian-Sheng Wu.

**Resources:** Jian-Sheng Wu.

**Conceptualization:** San-Ni Chen.

**Project administration:** San-Ni Chen.

**Writing – review & editing:** San-Ni Chen.
